# Human *Treponema pallidum* 11q/j isolate belongs to subsp. *endemicum* but contains two loci with a sequence in TP0548 and TP0488 similar to subsp. *pertenue* and subsp. *pallidum*, respectively

**DOI:** 10.1371/journal.pntd.0005434

**Published:** 2017-03-06

**Authors:** Lenka Mikalová, Michal Strouhal, Jan Oppelt, Philippe Alain Grange, Michel Janier, Nadjet Benhaddou, Nicolas Dupin, David Šmajs

**Affiliations:** 1 Department of Biology, Faculty of Medicine, Masaryk University, Brno, Czech Republic; 2 CEITEC–Central European Institute of Technology, Masaryk University, Brno, Czech Republic; 3 National Centre for Biomolecular Research, Masaryk University, Brno, Czech Republic; 4 Institut Cochin U1016, Laboratoire de Dermatologie—CNR Syphilis, Faculté de Médecine, Université Sorbonne Paris Descartes, Paris, France; 5 Centre des MST, Hôpital Saint-Louis, AP-HP, Paris, France; 6 Service de Bactériologie, Groupe Hospitalier Paris Centre Cochin-Hôtel Dieu-Broca, Paris, France; 7 Service de Dermatologie-Vénéréologie, Hôpital Cochin–Pavillon Tarnier, AP-HP, Paris, France; Institut Pasteur, FRANCE

## Abstract

**Background:**

*Treponema pallidum* subsp. *endemicum* (TEN) is the causative agent of endemic syphilis (bejel). An unusual human TEN 11q/j isolate was obtained from a syphilis-like primary genital lesion from a patient that returned to France from Pakistan.

**Methodology/Principal findings:**

The TEN 11q/j isolate was characterized using nested PCR followed by Sanger sequencing and/or direct Illumina sequencing. Altogether, 44 chromosomal regions were analyzed. Overall, the 11q/j isolate clustered with TEN strains Bosnia A and Iraq B as expected from previous TEN classification of the 11q/j isolate. However, the 11q/j sequence in a 505 bp-long region at the TP0488 locus was similar to *Treponema pallidum* subsp. *pallidum* (TPA) strains, but not to TEN Bosnia A and Iraq B sequences, suggesting a recombination event at this locus. Similarly, the 11q/j sequence in a 613 bp-long region at the TP0548 locus was similar to *Treponema pallidum* subsp. *pertenue* (TPE) strains, but not to TEN sequences.

**Conclusions/Significance:**

A detailed analysis of two recombinant loci found in the 11q/j clinical isolate revealed that the recombination event occurred just once, in the TP0488, with the donor sequence originating from a TPA strain. Since TEN Bosnia A and Iraq B were found to contain TPA-like sequences at the TP0548 locus, the recombination at TP0548 took place in a treponeme that was an ancestor to both TEN Bosnia A and Iraq B. The sequence of 11q/j isolate in TP0548 represents an ancestral TEN sequence that is similar to yaws-causing treponemes. In addition to the importance of the 11q/j isolate for reconstruction of the TEN phylogeny, this case emphasizes the possible role of TEN strains in development of syphilis-like lesions.

## Author summary

*Treponema pallidum* subsp. *endemicum* (TEN) is an uncultivable pathogenic treponeme that causes bejel (endemic syphilis), a chronic human infection mostly affecting children under 15 years of age, occurring mainly in several African and Middle East countries. In this work, we characterized a TEN 11q/j isolate from France that was obtained from an adult male with genital lesions, who was suspected of having syphilis and who received benzathine penicillin G. DNA sequencing of the isolate revealed two loci that were, rather than to TEN, related either to *T*. *pallidum* subsp. *pertenue* or to *T*. *pallidum* subsp. *pallidum* and likely resulted from recombination events. The recombination event in TP0488 as well as the recombination in TP0548, of the 11q/j, helped clarify the phylogeny of the TEN strains indicating that the recombination in TP0548 took place in a treponeme that was ancestral of Bosnia A and Iraq B, but was not an ancestor of the 11q/j isolate. In contrast, a recombination event in TP0488 appeared in the ancestor of the 11q/j isolate after separation of the ancestral treponeme of Bosnia A and Iraq B. This case also points to a possible role of TEN strains in development of syphilis-like lesions in countries with endemic syphilis.

## Introduction

*Treponema pallidum* subsp. *endemicum* (TEN) is the causative agent of bejel (endemic syphilis), a chronic human infection usually affecting children under 15 years of age. The primary stage of endemic syphilis is often localized to the mucosa of the oral cavity or nasopharynx and frequently remains undetected. Secondary lesions often mimic syphilitic lesions and are found on both mucosal and skin surfaces including the oral cavity, pharynx, and larynx (for review see [1]). The tertiary stage is characterized by gummatous or destructive lesions of mucosa, skin, and bones. Recently reported cases of bejel have come from African countries with dry climates including Mauretania, Niger, Chad, Mozambique and from countries in the Middle East including Turkey, Saudi Arabia, and Iran [1]. Moreover, several imported cases of bejel have been described in France [2] and Canada [3] in children coming from countries where endemic syphilis has been reported.

Compared to the syphilis-causing *Treponema pallidum* subsp. *pallidum* (TPA) and the yaws-causing *Treponema pallidum* subsp. *pertenue* (TPE) (reviewed in [4, 5]), TEN is the least well characterized and least studied human pathogenic treponeme. There are few genetic studies on TEN strains [3, 6–14], which is likely due to a limited number of available TEN samples. In fact, most studies on TEN strains described one of the two reference strains, i.e., Bosnia A or Iraq B. The Bosnia A strain was isolated in 1950 in southern Europe (Bosnia) from a 35-year old male with several mucosal and skin lesions [15], and the Iraq B strain was isolated in 1951 in Iraq from a 7-year old girl who had oral mucous lesions and an anal condylomata [15]. Because of the low number of available reference strains, only a single complete genome sequence of TEN Bosnia A has been published to date showing a close relatedness (higher than 99.9%) to TPE strains and several sequences surprisingly similar to TPA strains [16].

First reported in 2013, an unusual 11q/j subtype (defined by enhanced CDC typing) [17, 18] was found among samples taken from a syphilis patient in Paris [19] who had returned from Islamabad, Pakistan, where he admitted having had sex with commercial sex worker. Based on a partial sequence type reported for TP0548, Mikalová *et al*. [20] pointed out that this sequence was more related to the yaws-causing strains rather than to syphilis-causing strains. Further analyses resulted in the classification of the 11q/j isolate as a TEN treponeme [21].

In this study, we characterized the 11q/j isolate in a set of 44 independent chromosomal regions. Sequencing of these loci revealed that the 11q/j isolate belongs to the *T*. *pallidum* subsp. *endemicum* with two loci having sequences that were related to either TPE (TP0548) or TPA (TP0488). The relevance of these findings is discussed here.

## Materials and methods

### Ethics statement

The study was approved by the institutional review board of the Comité de Protection des Personnes d’Ile de France 3 (S.C.3005).

### Sample collection

The sample (a swab from an indurated genital ulceration) was collected from a 42-year-old heterosexual man who attended the outpatient STD clinic of Hôpital Saint-Louis (Paris) and was analyzed anonymously. Isolated DNA (20 μl) from this sample (referred as 11q/j) was obtained from the National Reference Center for Syphilis in France (CNR Syphilis, www.cnr-syphilis.fr) that had performed a routine analysis on DNA from clinical samples [22].

### DNA amplification and sequencing of the 11q/j isolate

A nested PCR protocol for detection of the *polA* gene was performed using the previously described outer primers, polA_outer_F1 (5´-TTCTGTGCTCACGTCTGGTC-3´) and polA_outer_R1 (5´-TGCAACCATCGTATCGAAAA-3´), which resulted in a 637 bp amplicon [23–25] and inner primers for nested *polA* PCR, polA_F1 (5´-TGCGCGTGTGCGAATGGTGTGGTC-3´) and polA_R1 (5´-CACAGTGCTCAAAAACGCCTGCACG-3´), resulting in a 377 bp amplicon, were used as described in Liu *et al*. [26]. This nested PCR protocol was shown to be able to detect 1–10 copies of treponemal DNA in a 1 μl of sample [23–25] and was used for detection of the number of treponemal genome equivalents in 1 μl of DNA. The original 11q/j DNA sample (3 μl) was randomly amplified using a REPLI-g Single Cell kit (Qiagen, Hilden, Germany) according to the manufacturer's instructions. Randomly amplified sample of 11q/j was then used for (i) direct nested PCR amplification with specific primers (listed in S1 Table) according to a previously published protocol [25, 27], (ii) whole DNA sequencing using an Illumina MiSeq Next-gen sequencer (Illumina, San Diego, CA, USA), and (iii) the subsequent amplification with *T*. *pallidum* specific primers used in the pooled segment genome sequencing (PSGS) method [16, 28–30], which was followed by Illumina sequencing.

### Sequencing of the selected loci of the TEN Iraq B strain

Regions successfully amplified from the 11q/j isolate were also amplified from another available DNA reference sample, i.e., TEN Iraq B. The TEN Iraq B DNA was provided by Dr. Kristin N. Harper from the Department of Population Biology, Ecology, and Evolution, Emory University, Atlanta, Georgia, USA, in 2005. The Iraq B DNA was amplified with PCR or the nested PCR protocol with the same specific primers used for nested PCR amplification of the 11q/j isolate (S1 Table).

### Gene annotation and comparison to other treponemal genomes

The obtained partial sequences from the 11q/j isolate and TEN Iraq B were either assembled from Sanger and/or Illumina sequencing reads using SeqMan or SegMan NGen software (DNASTAR, Madison, WI, USA), respectively, with default assembling parameters. Genes were annotated according to the whole genome sequence of TEN Bosnia A (CP007548.1; [16]) and the 11q/j isolate and the TEN Iraq B genes were tagged with TEND11qj_ and TENDIB_ prefixes, respectively. The resulting sequences of the 11q/j isolate and TEN Iraq B were analyzed and compared to the following genomes: TPA Nichols (CP004010.2; [31]), TPA SS14 (CP004011.1; [31]), TPE Samoa D (CP002374.1; [29]), TPE CDC-2 (CP002375.1; [29]), TPE Gauthier (CP002376.1; [29]), TPE Fribourg-Blanc (CP003902.1; [30]), and TEN Bosnia A [16]. Alignments of treponemal sequences were performed using SeqMan software and MEGA7 software [32]. Phylogenetic trees were constructed in MEGA7 software [32] using the Maximum Likelihood method based on the Tamura-Nei model [33].

### Assessment of probability of accumulation of random point mutations resembling recombination in TP0488 and TP0548 loci

The following formula was used to calculate the probability that the observed nucleotide sequences were caused by accumulation of individual mutations instead of a recombination: p_mut_ = (p_mut___gen_ x p_mut_nuc_)^n^, where p_mut_ = the end probability of mutations resembling recombinant events, p_mut_gen_ = the frequency of mutation per single nucleotide, p_mut_nuc_ = the probability of a nucleotide substitution into the nucleotide sequence in the putative recombinant region, n = the number of mutated nucleotides within the putative recombinant region. p_mut_gen_ was calculated based on the number of variable sites identified within all available sequences of the 11q/j sample (total length of 29,753 bp, except for loci TP0488 and TP0548) and the corresponding sequences of TEN Bosnia A and TEN Iraq B. p_mut_nuc_ had a constant value of 0.333 reflecting 3 possible substitutions changing the original sequence at each nucleotide site. Different probabilities of transitions and transversions were not considered in this analysis. The “n” was calculated based on the number of different nucleotide positions between the 11q/j isolate and one of the two TEN strains that matched either the TPA or TPE orthologous sequence.

### Accession numbers

The resulting sequences of the TEN 11q/j isolate and the TEN Iraq B with length ≥ 200 bp were deposited in the GenBank under following accession numbers: KY120774-KY120814 for TEN 11q/j isolate; KY120815-KY120855 for TEN Iraq B. The detailed overview of sequenced loci is shown in S2 Table.

## Results

### Amplification of the 11q/j DNA

The only available DNA-containing sample (20 μl) was obtained from the CNR Syphilis that performed the isolation of DNA from the original swab sample [22]. As revealed by the nested *polA* PCR reaction [23] with detection limit of less than 10 molecules [26], the sample contained undetectable amounts of treponemal DNA, i.e. less than 10 molecules of treponemal DNA per 1 μl. Following whole genome amplification with random primers, nested PCR protocol revealed positivity in a 10^−2^ dilution indicating, at least, 1x10^2^ copies of treponemal genome equivalents per 1 μl in a total of 50 μl of amplified sample. This randomly amplified sample was used for further analyses.

### Illumina sequencing of randomly and specifically amplified 11q/j DNA

**Fig 1:**
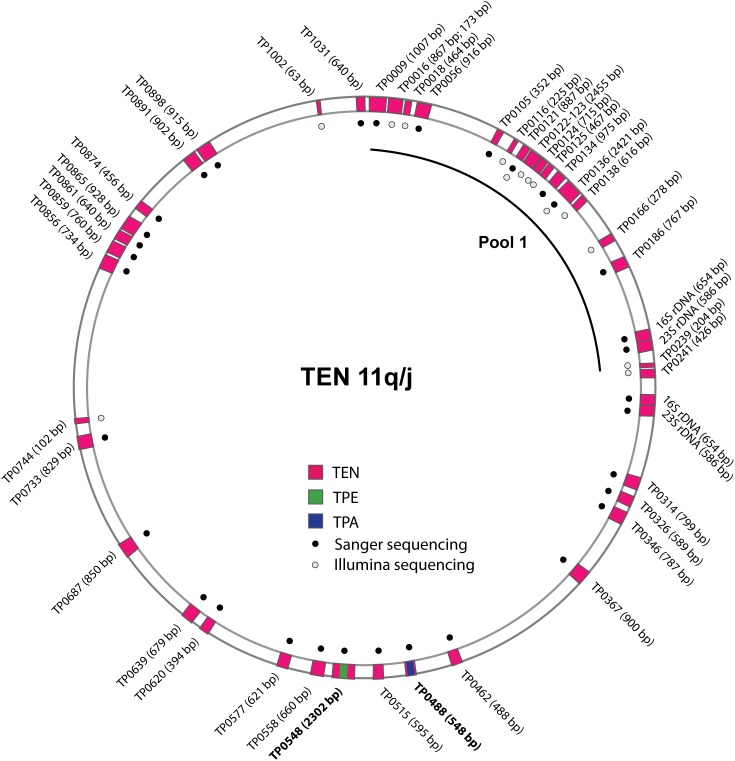
A schematic representation of the sequenced chromosomal loci of the TEN 11q/j isolate. The TEN 11q/j chromosome is shown as a circle and the pink regions represent sequenced chromosomal loci similar to corresponding chromosomal loci of TEN Bosnia A and Iraq B. The sequenced regions are not shown to scale, with respect to the whole genome length. The locus names of the sequenced loci are shown outside the circle together with the length of each sequenced region. Blue region within the TP0488 locus represents a sequence related to TPA treponemes, while the green region within the TP0548 locus represents a sequence related to TPE treponemes. Sanger sequenced regions are denoted as black circles and chromosomal loci sequenced using the Illumina method are shown as grey circles. The chromosomal regions amplified within Pool 1 of the PSGS sequencing approach are indicated by the black line.

The randomly amplified sample was used for direct Illumina sequencing and resulted in 1,786,712 individual reads. Of those, only 10 reads were mapped to the TEN Bosnia A genome indicating that the ratio of treponemal DNA to DNA from other species (mostly human) is less than 1:10^5^. Subsequently, the randomly amplified sample was used for specific amplification with the PSGS technique [16, 28–30] and primer pairs from Pool 1 amplifying the first quarter of the treponemal genome (Fig 1). Specific amplification resulted in a total of 353,006 individual reads, of which 41,308 reads were mapped to the TEN Bosnia A genome. Consensus sequences from at least 2 individual reads represented sequenced DNA regions of the 11q/j isolate. All regions determined by Illumina sequencing are shown in Fig 1 and S2 Table. Altogether, 15 genomic loci were obtained for the 11q/j isolate with lengths ranging from 63–2,455 bp, with total length of 9,626 bp and with coverage ranging from 2–15,832x.

### Nested PCR of randomly amplified 11q/j DNA

In addition to Illumina sequencing, a nested PCR of 31 chromosomal loci was performed from the randomly amplified 11q/j sample using 1 μl of the starting DNA template. The resulting amplicons were Sanger sequenced. Loci for nested PCR were selected based on whole genome comparisons of published TPE strains (Samoa D, CDC-2, Gauthier) and TEN Bosnia A. Preferentially, loci with accumulated single nucleotide variants (SNVs) and/or indels between TPE and TEN strains were selected as well as conservative genes suitable for unambiguous distinction between TPE and TEN subspecies. All regions amplified using nested PCR and sequenced using the Sanger method are presented in Fig 1 and S2 Table. The 16S and 23S rRNA loci were amplified from both genome positions [12]. The length of resulting sequences of the 11q/j isolate ranged from 352–2302 bp and represented a total of 23,979 bp.

### Sequenced genomic regions of the 11q/j isolate and TEN Iraq B strain

Illumina and Sanger sequencing of the 11q/j isolate resulted in sequences obtained from 44 chromosomal DNA regions covering, altogether, 32,635 bp (2.87%) of the TEN Bosnia A genome length (S2 Table). Two genomic regions within TP0121 and TP0136 genes, where both sequencing techniques partially overlapped, revealed identical sequences. The average length of sequenced regions in the 11q/j isolate was 742 bp (range 63–2,455 bp). The sequenced chromosomal regions were dispersed throughout the entire chromosome with distances ranging from 0.1–124.7 kb (Fig 1). All sequenced genomic regions of the 11q/j isolate were also amplified and Sanger sequenced from the TEN Iraq B DNA and these regions are described in S2 Table.

### The 11q/j sequence at the TP0488 locus

**Fig 2:**
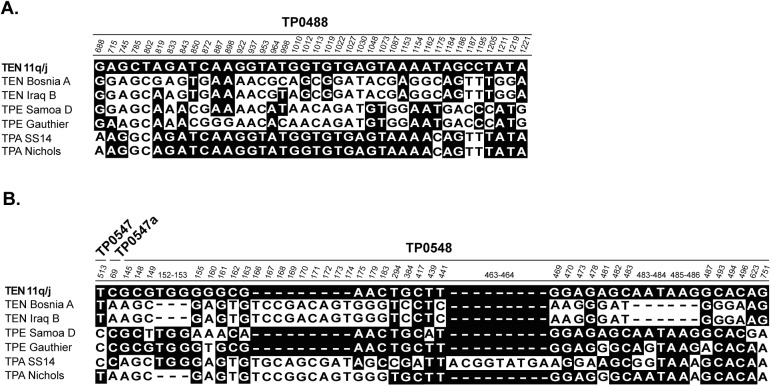
**Sequence alignments at the TEND11qj_0488 (A.) and TEND11qj_0548 (B.) loci with the orthologous sequences of the selected TEN, TPA, and TPE genomes.** Sequences of two TEN strains (Bosnia A and Iraq B), two TPE strains (Gauthier and Samoa D) and two TPA strains (Nichols and SS14) are shown. Numbers above the alignment represent gene coordinates in the corresponding genes of TEN Bosnia A (CP007548.1; [16]). The alignment of TEND11qj_0488 showed a sequence very similar to TPA strains, and TEND11qj_0548 was similar to TPE strains.

**Fig 3:**
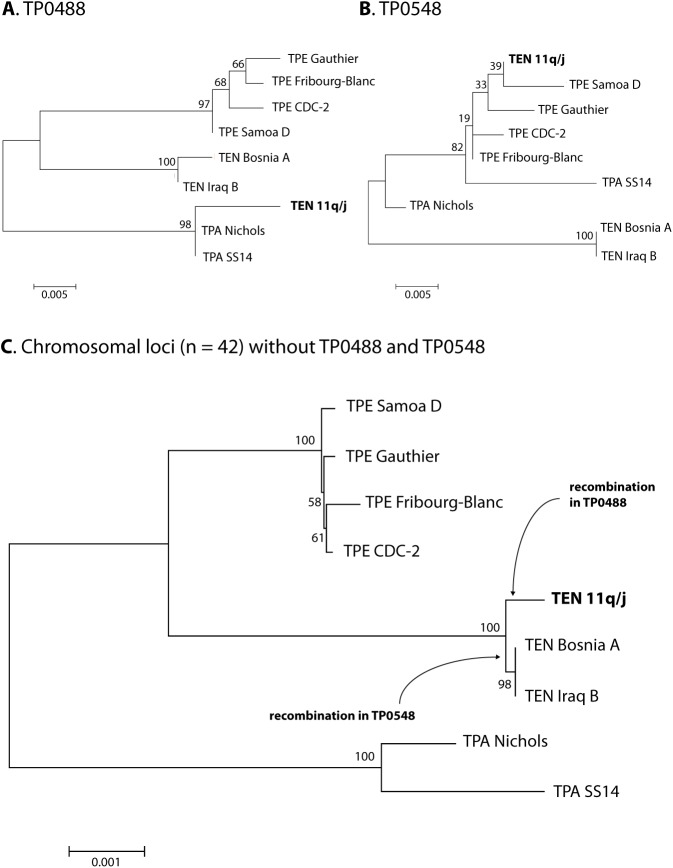
Unrooted tree based on the alignment of the TEND11qj_0488, TEND11qj_0548 and the remaining sequenced loci of the TEN 11q/j isolate. A. An unrooted tree was constructed from a 505 bp-long sequence of the locus TP0488, shown in Fig 2A between coordinates 715–1221 corresponding to TEN Bosnia A numbering (CP007548; [16]). B. An unrooted tree constructed from a 613 bp-long sequence of the locus TP0548, shown in Fig 2B between coordinates 69 of TENDBA_0547a and coordinate 623 of TENDBA_0548 (CP007548; [16]). C. An unrooted tree was constructed from concatenated sequences of 42 genomic regions of the 11q/j isolate, except for the TP0488 and TP0548 genes, and respective sequences of TPA strains (Nichols, SS14), TPE strains (CDC-2, Gauthier, Samoa D, and Fribourg-Blanc), and the TEN strains (Bosnia A, Iraq B) using the Maximum Likelihood method and MEGA7 software [31]. Bootstrap values based on 1,000 replications are shown next to the branches. All positions containing indels in at least one genome sequence were omitted from the analysis. There was a total of 29,447 nucleotide positions aligned in the final dataset. The bar scale corresponds to a difference of 0.005 (A.), 0.005 (B.) and 0.001 (C.) nucleotides. While the 11q/j isolate cluster in the TP0488 locus with TPA strains and in the TP0548 locus with TPE strains, a tree constructed from concatenated sequences of 42 genomic regions of the 11q/j isolate cluster with TEN strains. A position within the tree where the putative recombination events, involving TP0488 and TP0548, likely occurred is shown, respectively.

Interestingly, sequencing of a short gene fragment (548 bp) of TENDBA_0488 between positions 684–1231 revealed that the sequence of the 11q/j isolate was very similar to the sequence in TPA Nichols, but not to TEN strains (Fig 2A); suggesting the occurrence of a recombination event at this locus. The minimal size of recombinant DNA sequence was 505 nucleotides (between coordinates 715–1219; Fig 2A). A set of 21 nucleotide positions of the 11q/j isolate were different from TEN Bosnia A as well at TEN Iraq B, but identical to TPA strains. A partial sequence of the TEND11qj_0488 from the 11q/j isolate, representing the recombinant part (505 bp long fragment), was used for construction of a tree (Fig 3A) that revealed clustering of the 11q/j isolate within TPA strains, not within TEN strains. The probability that the observed nucleotide sequence within this locus was caused by an accumulation of individual mutations instead of a recombination was tested using the following formula: p_mut_ = (p_mut___gen_ x p_mut_nuc_)^n^ (see Materials and Methods). p_mut_gen_ was calculated based on the number of variable sites identified within all available sequences of the 11q/j sample (22 variable positions in a total length of 29,753 bp from the 3 analyzed TEN genomes; 0.00074 nt differences per 1 bp). Loci TP0488 and TP0548 were not included in this calculation. The “n” was calculated based on the number of variable positions detected in the sequence alignment presented in Fig 2. In the TP0488 gene, there was a total of 23 nucleotide positions in the 11q/j sequence that differed from TEN strain Bosnia A but were identical to TPA strain SS14. With the assumption that the 11q/j isolate represents a TEN strain, the probability that the accumulated SNVs within the TP0488 of the 11q/j isolate were due to accumulation of individual mutations would be: p_mut_ = (0,00074 x 0.333)^23^, i.e. p_mut_ = 1.01987 x 10^−83^.

To rule out potential co-infection with TPA and TEN in this patient, Illumina sequencing reads of the 11q/j sample, especially in regions with positions that differ between TPA and TEN, were evaluated and revealed 20 informative sites with coverage ≥ 4x (range 4x–94x). However, there was no heterogeneity in these positions, excluding co-infection with multiple strains.

### The 11q/j sequence at the TP0548 locus

As shown previously, the 11q/j isolate within its 86 bp-long fragment of TP0548 gene revealed a new sequence type that is, in fact, related to TPE strains [20]. Analysis of a larger 2,302 bp-long region comprising TP0547, TP0547a, and TP0548 genes (positions 589,926–592,227 corresponding to the whole genome sequence of the TEN Bosnia A) revealed that the sequence of the 11q/j isolate, within the TP0548 gene, was very similar to TPE strains, especially to a sequence from TPE Samoa D (Figs 2B and 3B). The minimal size of recombinant DNA sequence was 613 nucleotides (between coordinate 69 of the TENDBA_0547a and coordinate 623 of the TENDBA_0548) and comprised 56 variable positions (Fig 2B). Thirty-seven of the nucleotide positions of the 11q/j isolate were different from TEN Bosnia A and TEN Iraq B, but identical to at least one of the TPE Samoa D or Gauthier strains (Fig 2B). Both TEN Bosnia A and Iraq B showed 23 nucleotide positions identical to at least one of the TPA strains, i.e., to Nichols or SS14, but different from the 11q/j isolate. A partial sequence of TEND11qj_0548 (613 bp) was used for construction of a tree (Fig 3B) and reveled clustering of the 11q/j isolate among TPE strains, but not among TEN strains. The probability that the observed nucleotide sequence within this locus was caused by an accumulation of individual mutations instead of a recombination was p_mut_ = 1.12245 x 10^−65^ (p_mut_ = (0.00074 x 0.333)^18^), since there was a total of 18 SNVs that differed from TEN strain Bosnia A but were identical to TPE strain Gauthier. Indels were omitted from the calculation.

### Sequence relatedness of remaining chromosomal loci of the 11q/j isolate to other treponemal genomes

The sequences of 42 chromosomal regions, excluding TP0488 and TP0548 sequences, were concatenated and used to construct a phylogenetic tree to visualize the relatedness of 11q/j isolate to other treponemal genomes (Fig 3C). The corresponding genome regions from the published whole genome sequences of two TPA strains (Nichols, SS14), four TPE strains (CDC-2, Gauthier, Samoa D, and Fribourg-Blanc), and TEN Bosnia A were used. Moreover, the dataset was supplemented with sequences of TEN Iraq B. All positions in the alignment containing gaps and missing data were eliminated resulting in a total of 29,447 positions in the final dataset having 509 variable sites. Overall, the 11q/j isolate clustered with both TEN Bosnia A and TEN Iraq B, indicating that most chromosomal loci of the11q/j isolate were consistent with TEN classification.

## Discussion

In this work, we analyzed an interesting human clinical isolate, 11q/j, that was first reported in 2013 as a case of syphilis [19], but due to an unusual sequence pattern at the TP0548 locus, similar to TPE, it was thought to be an imported case of yaws [20]. In 2016, the 11q/j isolate was further characterized in 7 genomic loci and classified as subspecies TEN [23]. Due to the unusual syphilis-yaws-bejel history of the 11q/j isolate we characterized larger genome regions of this clinical sample using different sequencing approaches.

The small amount of treponemal DNA within the only available sample of the 11q/j isolate (copy number less than 10 molecules of treponemal DNA per 1 μl) with an excessive amount of contaminating human DNA, which exceeded the treponemal DNA by at least 100,000 times, precluded the use of other techniques that have been recently reported to be effective in sequencing treponemal DNA directly from clinical samples [34, 35]. Efficient enrichment of treponemal DNA requires the number of treponemal copies > 1x10^4^ per 1 μl [34]. In fact, enrichment of the TEN Iraq B DNA sample, containing 10^4^ copies per 1 μl, revealed genome coverage less than 12.4% [35]. For these reasons, we mostly used nested PCR in this study. In all cases, amplification was done from samples containing at least 10^2^ copies of treponemal DNA to avoid introduction of sequencing errors.

As reported in a previous study based on analysis of 7 chromosomal regions, classification of the clinical isolate 11q/j was consistent with *T*. *pallidum* subsp. *endemicum* [21]. In this work, we confirmed this finding based on analyses of 42 chromosomal regions (excluding the TP0488 and TP0548 loci), which were independently amplified and analyzed. The corresponding phylogenetic tree revealed a clear clustering of the 11q/j isolate with TEN strains (Fig 3C). In addition, the genetic distance between the 11q/j isolate and TEN Bosnia A and Iraq B was greater than the distance between Bosnia A and Iraq B, indicating that the ancestor of the 11q/j isolate diverged before TEN Bosnia A and TEN Iraq B diversified.

Although the sequenced portion of the 11q/j isolate represented less than 3% of the total genome length, the number of analyzed nucleotide positions informative for differentiation between TPE and TEN was much larger. Considering the extent of similarity of the genome sequences of available TPE and TEN strains (i.e., they are 99.91–99.94% similar), there were relatively few (711–970) variable sites between TEN Bosnia A and TPE strains Gauthier, CDC-2 and Samoa D [16]. Within the 11q/j isolate, 196 (20–28%) of these variable sites were sequenced and 98% of them revealed sequence similarity to TEN strains. Therefore, it is very likely that the classification of 11qj isolate as TEN strain will remain the same even after acquisition of additional genomic sequences.

Sequence analysis of TP0488 of the 11q/j sample revealed a sequence very similar to TPA strains. A similar situation has been previously reported in the genome of Bosnia A, where several chromosomal regions including TP0326, TP0488, TP0577, TP0858, TP0968, and TP1031 showed striking similarity to TPA treponemes [16]. However, the TPA-like sequences at the TP0488 locus of Bosnia A and Iraq B strains were different from the TP0488 sequence of the 11q/j sample and were located between positions 1175–1195 (Fig 2A), indicating that the 11q/j recombination event was independent of the recombination event at the TP0488 locus in the ancestor of TEN Bosnia A and TEN Iraq B. Interestingly, in the TPA Mexico A, TP0488 was found to contain a sequence very similar to that found in TPE strains, suggesting that the TP0488 locus is prone to gene recombination [36]. The TP0488 gene encodes a methyl-accepting chemotaxis protein (Mcp2-1) [37] and, as shown by expression profiling of treponemes isolated from rabbit infections, is highly expressed in TPA strains [38]. Moreover, the Mcp2-1 protein has been shown to elicit a humoral response [37]. In the TPA Mexico A genome, 8 out of 18 TPE-like changes were located in the Cache domain (domain binding small molecules) [39]. Similarly, 13 out of 21 amino acid replacements resulting from recombination in the 11q/j isolate were also located in the Cache domain, suggesting differences in binding properties of the Mcp2-1 protein. As discussed in a previous work [36], the observed sequence patterns are consistent with recombination events that have likely occurred during parallel human infections with both TPA and TEN or TPA and TPE treponemes.

Gene TP0548, on the other hand, was for the first time found to be recombinant. TP0548 from the 11q/j isolate appeared to be composed of sequences (in addition to TEN sequences) originating from TPE treponemes. Moreover, TEN Bosnia A and TEN Iraq B showed TPA-like sequences within this locus. For this reason, as well as the fact that the ancestor of the 11q/j isolate diverged before the ancestor of TEN Bosnia A and Iraq B strains, it is more plausible that the recombination event occurred in the common ancestor of Bosnia A and Iraq B rather than in the 11q/j isolate. The divergence of the ancestor of the 11q/j isolate before the ancestor of TEN Bosnia A and Iraq B strains is supported by greater genetic distances between the 11q/j isolate and both TEN strains (Bosnia A, Iraq B) compared to distances between Bosnia A and Iraq B (Fig 3). According to this scenario, the recombination occurred in a TEN strain that was ancestral to both the Bosnia A and Iraq B, which incorporated the TPA sequence into this locus (Fig 3C). The sequence of the 11q/j isolate thus represents the original TEN sequence that is similar to TPE strains. The TP0548 was predicted to encode for a rare outer membrane protein [40] and, as shown by molecular typing studies, is highly variable among syphilis isolates [18, 22, 25, 27]. The tendency of this locus to recombine, although shown only in TEN, should be considered during interpretation of data from both enhanced CDC and sequencing-based typing of syphilis-causing strains.

The calculated probability that the observed SNVs within TP0488 and TP0548 were caused by random mutations was extremely low, i.e. 1.01987 x 10^−83^ and 1.12245 x 10^−65^, respectively, suggesting that recombination occurred in these loci. Since mutation frequency per 1 bp within the TEN subspecies was calculated based on the sequences obtained for the 11q/j isolate, there was a potential bias in preferential sequencing of TEN variable regions. However, inclusion of additional chromosomal loci would likely lower the final probability even more. Moreover, the calculated SNV density in TEN strains (0.74 nt per 1000 bp) differed only slightly compared to densities within other treponemal subspecies (0.36 nt per 1000 bp in TPA; 0.14 nt per 1000 bp in TPE; [29]).

Both intra-genomic and inter-genomic recombination events have been identified in uncultivable pathogenic treponemes and are summarized in Table 1. While intra-genomic homologous recombination have been found in *tpr* genes [7, 10, 41, 42], several inter-genomic recombination events have already been described in the literature [16, 36].

**Table 1:** A to-date overview of recombinant genes in pathogenic treponemes.

Gene #	Gene name	Definition	Recombination type	Reference
TP0117	*tprC*	Tpr protein C	Intra-strain	[7]
TP0131	*tprD*	Tpr protein D	Intra-strain	[7]
TP0133		hypothetical protein	Inter-strain	[16]
rDNA loci		tRNA spacer	Intra-strain	[12]
TP0316	*tprG*	Tpr protein G	Intra-strain	[7]
TP0326	*tp92*	BamA ortologue and rare outer membrane protein	Inter-strain	[9, 16, 36]
TP0488	*mcp2*	methyl-accepting chemotaxis protein	Inter-strain	[16, 36, This study]
TP0548		putative membrane protein	Inter-strain	This study
TP0577		putative membrane protein	Inter-strain	[16]
TP0620	*tprI*	Tpr protein I	Intra-strain	[7]
TP0621	*tprJ*	Tpr protein J	Intra-strain	[7]
TP0858		putative lipoprotein	Inter-strain	[16]
TP0897	*tprK*	Tpr protein K	Intra-strain	[10, 41, 42]
TP0968		hypothetical protein	Inter-strain	[16]
TP1031	*tprL*	Tpr protein L	Inter-strain	[16]

The fact that the infection caused by the TEN 11q/j isolate resembled early syphilis with lesions located on the genitals supports previous findings that both TPA and TEN strains form similar, clinically undiscernible primary lesions. In a similar case, TEN Bosnia A was isolated from genital lesions of a 35-year old male, although in this case, lesions were also found in the oral cavity and pharynx and the patient showed secondary lesions on the face, trunk and extremities. The patient which was the source of the 11q/j isolate in this study, reported that he returned to France from Islamabad, Pakistan, where he admitted having had sexual contact with commercial sex worker. Since Pakistan is located close to countries that have recently reported cases of endemic syphilis, including Saudi Arabia and Iran [1], such an infection should not be surprising. Given the known number of repetitions in the *arp* gene, the restriction pattern of the amplified *tprEGJ* genes, and the TP0548 sequence, the enhanced CDC genotype [18] can be deduced for TEN strains. For TEN Bosnia A and Iraq B, the 10q/c and 8q/c genotypes can be predicted based on published data, respectively [8, 16, 20]. Interestingly, similar subtypes have already been identified among tested clinical isolates from China including 9h/c, 10h/c, and 9o/c [43]. In fact, the electrophoretic *tpr* pattern “h” differs from the pattern “q” by one fragment (i.e., 804 bp in pattern “h” vs. 726 bp in “q”) and “o” differs from pattern “q” by the absence of a 315 bp fragment [44]. Close similarity of identified subtypes of human syphilis isolates to predicted subtypes of TEN Bosnia A and TEN Iraq B suggests that TEN strains could and should be sporadically detected among human samples from patients suspected of having syphilis. In such situations, suspicious samples should be further analyzed to obtain an unequivocal classification of either a TPA strain or a TEN strain.

Taken together, analysis of the 11q/j isolate revealed a TEN genome seemingly containing two recombination events and highlights the fact that TEN strains could cause syphilis-like lesions in humans. A more detailed analysis revealed that the 11q/j isolate had just one recombinant locus, TP0488. The recombination of TP0548 took place in a treponeme that was the ancestor of both TEN Bosnia A and TEN Iraq B.

## Supporting information

S1 Table List of primers used in this study. (XLSX)

S2 Table List of sequenced regions of the TEN 11q/j isolate and TEN Iraq B strain. (XLSX)

## References

[1] Giacani L, Lukehart SA. The endemic treponematoses. Clin Microbiol Rev. 2014;27(1): 89–115. 10.1128/CMR.00070-13 24396138 PMC3910905

[2] Vabres P, Roose B, Berdah S, Fraitag S, Prost YD. Bejel: an unusual cause of stomatitis in the child. Ann Dermatol Venereol. 1999;126(1): 49–50. 10095894

[3] Fanella S, Kadkhoda K, Shuel M, Tsang R. Local transmission of imported endemic syphilis, Canada, 2011. Emerging Infect Dis. 2012;18(6): 1002–1004. 10.3201/eid1806.111421 22607961 PMC3358156

[4] Šmajs D, Norris SJ, Weinstock GM. Genetic diversity in *Treponema pallidum*: implications for pathogenesis, evolution and molecular diagnostics of syphilis and yaws. Infect Genet Evol. 2012;12(2): 191–202. 10.1016/j.meegid.2011.12.001 22198325 PMC3786143

[5] Radolf JD, Deka RK, Anand A, Šmajs D, Norgard MV, Yang XF. *Treponema pallidum*, the syphilis spirochete: making a living as a stealth pathogen. Nat Rev Microbiol. 2016; 10.1038/nrmicro.2016.141 PMC5106329 27721440

[6] Centurion-Lara A, Arroll T, Castillo R, Shaffer JM, Castro C, Van Voorhis WC, et al Conservation of the 15-kilodalton lipoprotein among *Treponema pallidum* subspecies and strains and other pathogenic treponemes: genetic and antigenic analyses. Infect Immun. 1997;65(4): 1440–1444. 9119485 10.1128/iai.65.4.1440-1444.1997 PMC175151

[7] Gray RR, Mulligan CJ, Molini BJ, Sun ES, Giacani L, Godornes C, et al Molecular evolution of the tprC, D, I, K, G, and J genes in the pathogenic genus *Treponema*. Mol Biol Evol. 2006;23(11): 2220–2233. 10.1093/molbev/msl092 16926243

[8] Harper KN, Liu H, Ocampo PS, Steiner BM, Martin A, Levert K, et al The sequence of the acidic repeat protein (*arp*) gene differentiates venereal from nonvenereal *Treponema pallidum* subspecies, and the gene has evolved under strong positive selection in the subspecies that causes syphilis. FEMS Immunol Med Microbiol. 2008;53(3): 322–332. 10.1111/j.1574-695X.2008.00427.x 18554302

[9] Harper KN, Ocampo PS, Steiner BM, George RW, Silverman MS, Bolotin S, et al On the origin of the treponematoses: a phylogenetic approach. PLoS Negl Trop Dis. 2008;2(1): e148 10.1371/journal.pntd.0000148 18235852 PMC2217670

[10] Giacani L, Brandt SL, Puray-Chavez M, Reid TB, Godornes C, Molini BJ, et al Comparative investigation of the genomic regions involved in antigenic variation of the TprK antigen among treponemal species, subspecies, and strains. J Bacteriol. 2012;194(16): 4208–4225. 10.1128/JB.00863-12 22661689 PMC3416249

[11] Centurion-Lara A, Giacani L, Godornes C, Molini BJ, Brinck Reid T, Lukehart SA. Fine analysis of genetic diversity of the *tpr* gene family among treponemal species, subspecies and strains. PLoS Negl Trop Dis. 2013;7(5): e2222 10.1371/journal.pntd.0002222 23696912 PMC3656149

[12] Čejková D, Zobaníková M, Pospíšilová P, Strouhal M, Mikalová L, Weinstock GM, et al Structure of *rrn* operons in pathogenic non-cultivable treponemes: sequence but not genomic position of intergenic spacers correlates with classification of *Treponema pallidum* and *Treponema paraluiscuniculi* strains. J Med Microbiol. 2013;62(Pt 2): 196–207. 10.1099/jmm.0.050658-0 23082031 PMC3755535

[13] Nechvátal L, Pětrošová H, Grillová L, Pospíšilová P, Mikalová L, Strnadel R, et al Syphilis-causing strains belong to separate SS14-like or Nichols-like groups as defined by multilocus analysis of 19 *Treponema pallidum* strains. Int J Med Microbiol. 2014;304(5–6): 645–653. 10.1016/j.ijmm.2014.04.007 24841252

[14] Čejková D, Strouhal M, Norris SJ, Weinstock GM, Šmajs D. A Retrospective Study on Genetic Heterogeneity within *Treponema* Strains: Subpopulations Are Genetically Distinct in a Limited Number of Positions. PLoS Negl Trop Dis. 2015;9(10): e0004110 10.1371/journal.pntd.0004110 26436423 PMC4593590

[15] Turner TB, Hollander DH. Biology of the treponematoses based on studies carried out at the International Treponematosis Laboratory Center of the Johns Hopkins University under the auspices of the World Health Organization. Monogr Ser World Health Organ. 1957;(35): 3–266. 13423342

[16] Štaudová B, Strouhal M, Zobaníková M, Čejková D, Fulton LL, Chen L, et al Whole genome sequence of the *Treponema pallidum* subsp. *endemicum* strain Bosnia A: the genome is related to yaws treponemes but contains few loci similar to syphilis treponemes. PLoS Negl Trop Dis. 2014;8(11): e3261 10.1371/journal.pntd.0003261 25375929 PMC4222731

[17] Pillay A, Liu H, Chen CY, Holloway B, Sturm AW, Steiner B, et al Molecular subtyping of *Treponema pallidum* subspecies *pallidum*. Sex Transm Dis. 1998;25(8): 408–414. 9773432 10.1097/00007435-199809000-00004

[18] Marra CM, Sahi SK, Tantalo LC, Godornes C, Reid T, Behets F, et al Enhanced molecular typing of *Treponema pallidum*: geographical distribution of strain types and association with neurosyphilis. J Infect Dis. 2010;202(9): 1380–1338. 10.1086/656533 20868271 PMC3114648

[19] Grange PA, Allix-Beguec C, Chanal J, Benhaddou N, Gerhardt P, Morini J-P, et al Molecular subtyping of *Treponema pallidum* in Paris, France. Sex Transm Dis. 2013;40(8): 641–644. 10.1097/OLQ.0000000000000006 23859911

[20] Mikalová L, Strouhal M, Grillová L, Šmajs D. The molecular typing data of recently identified subtype 11q/j of *Treponema pallidum* subsp. *pallidum* suggest imported case of yaws. Sex Transm Dis. 2014;41(9): 552–553. 10.1097/OLQ.0000000000000165 25118969

[21] Grange PA, Mikalová L, Gaudin C, Strouhal M, Janier M, Benhaddou N, et al *Treponema pallidum* 11qj Subtype May Correspond to a *Treponema pallidum* Subsp. *Endemicum* Strain. Sex Transm Dis. 2016;43(8): 517–518. 10.1097/OLQ.0000000000000474 27419817

[22] Grange PA, Gressier L, Dion PL, Farhi D, Benhaddou N, Gerhardt P, et al Evaluation of a PCR test for detection of *Treponema pallidum* in swabs and blood. J Clin Microbiol 2012; 50:546–552. 10.1128/JCM.00702-11 22219306 PMC3295187

[23] Flasarová M, Šmajs D, Matejková P, Woznicová V, Heroldová-Dvořáková M, Votava M. Molecular detection and subtyping of Treponema pallidum subsp. pallidum in clinical specimens. Epidemiol Mikrobiol Imunol. 2006;55(3): 105–111. 16970074

[24] Woznicová V, Šmajs D, Wechsler D, Matĕjková P, Flasarová M. Detection of *Treponema pallidum* subsp. *pallidum* from skin lesions, serum, and cerebrospinal fluid in an infant with congenital syphilis after clindamycin treatment of the mother during pregnancy. J Clin Microbiol. 2007;45(2): 659–661. 10.1128/JCM.02209-06 17151205 PMC1829021

[25] Flasarová M, Pospíšilová P, Mikalová L, Vališová Z, Dastychová E, Strnadel R, et al Sequencing-based molecular typing of *Treponema pallidum* strains in the Czech Republic: all identified genotypes are related to the sequence of the SS14 strain. Acta Derm Venereol. 2012;92(6): 669–674. 10.2340/00015555-1335 22434073

[26] Liu H, Rodes B, Chen CY, Steiner B. New tests for syphilis: rational design of a PCR method for detection of *Treponema pallidum* in clinical specimens using unique regions of the DNA polymerase I gene. J Clin Microbiol. 2001;39(5): 1941–1946. 10.1128/JCM.39.5.1941-1946.2001 11326018 PMC88053

[27] Grillová L, Pĕtrošová H, Mikalová L, Strnadel R, Dastychová E, Kuklová I, et al Molecular typing of *Treponema pallidum* in the Czech Republic during 2011 to 2013: increased prevalence of identified genotypes and of isolates with macrolide resistance. J Clin Microbiol. 2014;52: 3693–3700. 10.1128/JCM.01292-14 25100820 PMC4187743

[28] Weinstock GM, Šmajs D, Hardham J, Norris SJ. From microbial genome sequence to applications. Res Microbiol. 2000;151(2): 151–158. 10865961 10.1016/s0923-2508(00)00115-7

[29] Čejková D, Zobaníková M, Chen L, Pospíšilová P, Strouhal M, Qin X, et al Whole genome sequences of three *Treponema pallidum* ssp. *pertenue* strains: yaws and syphilis treponemes differ in less than 0.2% of the genome sequence. PLoS Negl Trop Dis. 2012;6(1): e1471 10.1371/journal.pntd.0001471 22292095 PMC3265458

[30] Zobaníková M, Strouhal M, Mikalová L, Čejková D, Ambrožová L, Pospíšilová P, et al Whole genome sequence of the *Treponema* Fribourg-Blanc: unspecified simian isolate is highly similar to the yaws subspecies. PLoS Negl Trop Dis. 2013;7(4): e2172 10.1371/journal.pntd.0002172 23638193 PMC3630124

[31] Pětrošová H, Pospíšilová P, Strouhal M, Čejková D, Zobaníková M, Mikalová L, et al Resequencing of *Treponema pallidum* ssp. *pallidum* strains Nichols and SS14: correction of sequencing errors resulted in increased separation of syphilis treponeme subclusters. PLoS ONE. 2013;8(9): e74319 10.1371/journal.pone.0074319 24058545 PMC3769245

[32] Kumar S, Stecher G, Tamura K. MEGA7: Molecular Evolutionary Genetics Analysis version 7.0 for bigger datasets. Mol Biol Evol. 2016;33(7): 1870–1874. 10.1093/molbev/msw054 27004904 PMC8210823

[33] Tamura K, Nei M. Estimation of the number of nucleotide substitutions in the control region of mitochondrial DNA in humans and chimpanzees. Mol Biol Evol. 1993;10: 512–526. 8336541 10.1093/oxfordjournals.molbev.a040023

[34] Pinto M, Borges V, Antelo M, Pinheiro M, Nunes A, Azevedo J, et al Genome-scale analysis of the non-cultivable *Treponema pallidum* reveals extensive within-patient genetic variation. Nat Microbiol. 2016;2: 16190 10.1038/nmicrobiol.2016.190 27748767

[35] Arora N, Schuenemann VJ, Jäger G, Peltzer A, Seitz A, Herbig A, et al Origin of modern syphilis and emergence of a pandemic *Treponema pallidum* cluster. Nature Microbiology. 2016; Forthcoming 10.1038/nmicrobiol.2016.245 27918528

[36] Pětrošová H, Zobaníková M, Čejková D, Mikalová L, Pospíšilová P, Strouhal M, et al Whole genome sequence of *Treponema pallidum* ssp. *pallidum*, strain Mexico A, suggests recombination between yaws and syphilis strains. PLoS Negl Trop Dis. 2012;6(9): e1832 10.1371/journal.pntd.0001832 23029591 PMC3447947

[37] Greene SR, Stamm LV. Molecular characterization of *Treponema pallidum mcp2*, a putative chemotaxis protein gene. Infect Immun. 1998;66(6): 2999–3002. 9596781 10.1128/iai.66.6.2999-3002.1998 PMC108303

[38] Šmajs D, McKevitt M, Howell JK, Norris SJ, Cai W-W, Palzkill T, et al Transcriptome of *Treponema pallidum*: gene expression profile during experimental rabbit infection. J Bacteriol. 2005;187(5): 1866–1874. 10.1128/JB.187.5.1866-1874.2005 15716460 PMC1063989

[39] Anantharaman V, Aravind L. Cache—a signaling domain common to animal Ca(2+)-channel subunits and a class of prokaryotic chemotaxis receptors. Trends Biochem Sci. 2000;25(11): 535–537. 11084361 10.1016/s0968-0004(00)01672-8

[40] Cox DL, Luthra A, Dunham-Ems S, Desrosiers DC, Salazar JC, Caimano MJ, et al Surface immunolabeling and consensus computational framework to identify candidate rare outer membrane proteins of *Treponema pallidum*. Infect Immun. 2010;78(12): 5178–5194. 10.1128/IAI.00834-10 20876295 PMC2981305

[41] Centurion-Lara A, LaFond RE, Hevner K, Godornes C, Molini BJ, Van Voorhis WC, et al Gene conversion: a mechanism for generation of heterogeneity in the tprK gene of *Treponema pallidum* during infection. Mol Microbiol. 2004;52(6): 1579–1596. 10.1111/j.1365-2958.2004.04086.x 15186410

[42] Giacani L, Molini BJ, Kim EY, Godornes BC, Leader BT, Tantalo LC, et al Antigenic variation in *Treponema pallidum*: TprK sequence diversity accumulates in response to immune pressure during experimental syphilis. J Immunol. 2010;184(7): 3822–3829. 10.4049/jimmunol.0902788 20190145 PMC3042355

[43] Peng R-R, Yin Y-P, Wei W-H, Wang H-C, Zhu B-Y, Liu Q-Z, et al Molecular typing of *Treponema pallidum* causing early syphilis in China: a cross-sectional study. Sex Transm Dis. 2012;39(1): 42–45. 10.1097/OLQ.0b013e318232697d 22183845

[44] Pillay A. Treponema In: Filippis I, McKee ML, eds. Molecular typing in bacterial infections. Humana Press 2013: 311–326.

